# Effects of kinematic vibrotactile feedback on learning to control a virtual prosthetic arm

**DOI:** 10.1186/s12984-015-0025-5

**Published:** 2015-03-24

**Authors:** Christopher J Hasson, Julia Manczurowsky

**Affiliations:** Neuromotor Systems Laboratory, Department of Physical Therapy, Movement and Rehabilitation Sciences, Northeastern University, 360 Huntington Avenue, 301 Robinson Hall, Boston, MA 02115-5005 USA

**Keywords:** Vibrotactile feedback, Motor learning, Motor control, Prosthetics, Virtual reality, Myoelectric

## Abstract

**Background:**

After a limb is lost a prosthesis can restore function. For maximum utility, prosthetic limbs should accept movement commands and provide force and motion feedback, which can be conveyed with vibrotactile feedback (VIBF). While prior studies have shown that force-based VIBF benefits control, the merits of motion-based VIBF are unclear. Our goal was to clarify the effectiveness of position- and velocity-based VIBF for prosthetic arm control.

**Methods:**

Healthy adults with normal limb function practiced a goal-directed task with a virtual myoelectric prosthetic arm. A linear resonant actuator on the wrist provided VIBF. Two groups with nine subjects each received amplitude modulated VIBF in addition to visual feedback while practicing the task. In one group, the VIBF was proportional to the virtual arm’s position, and in the other group, velocity. A control group of nine subjects received only visual feedback. Subjects practiced for 240 trials, followed by 180 trials with feedback manipulations for the VIBF groups. Performance was characterized by end-point error, movement time, and a composite skill measure that combined these quantities. A second experiment with a new group of five subjects assessed discrimination capabilities between different position- and velocity-based VIBF profiles.

**Results:**

With practice all groups improved their skill in controlling the virtual prosthetic arm. Subjects who received additional position- and velocity-based VIBF learned at the same rate as the control group, who received only visual feedback (learning rate time constant: about 40 trials). When visual feedback was subsequently removed leaving only VIBF, performance was no better than with no feedback at all. When VIBF was removed leaving only visual feedback, about half of the participants performed better, instead of worse. The VIBF discrimination tests showed that subjects could detect virtual arm angular position and velocity differences of about 5 deg and 20 deg/s, respectively.

**Conclusions:**

Kinematic VIBF did not increase the rate of skill acquisition or improve performance when controlling a virtual myoelectric prosthetic arm, whether provided in isolation or coupled with visual feedback. VIBF had a deleterious effect on performance for some individuals, who may have had difficulty integrating kinematic VIBF information into their control strategies.

## Background

After a limb is lost through injury or disease, a prosthesis can be used to restore function. For maximum utility, prosthetic limbs should accept movement commands from the user and provide proprioceptive feedback. Proprioception refers to information about the position and velocity of the body segments (kinematics), and forces exerted on and between body segments (kinetics). In humans, this information is normally provided by various receptors, including muscle spindles, Golgi tendon organs, joint receptors, and cutaneous mechanoreceptors [1-3]. Without proprioception, a prosthesis user must rely on visual information to determine prosthesis position and velocity, and information about prosthesis-environment interaction forces can only be obtained through the limb-prosthesis connection. Consequently, motor function with a prosthetic limb remains below that of a natural limb [4].

Artificial prosthesis proprioceptive information can be supplied via direct stimulation of intact sensory afferents [5-7]. An alternative is to use sensory substitution, such as vibrotactile feedback (VIBF), which is a relatively cheap and non-invasive method of providing artificial proprioception [8]. These properties make the use of VIBF attractive, as this technology can be quickly incorporated into prosthetic devices [9]. Typically, VIBF is delivered by one or more small linear resonant actuators placed on the surface of the skin. Past studies have focused on using VIBF to provide information about human-environment contact forces because force information cannot be obtained by visual inspection (although predictions can be made on the basis of prior experience [10]). Providing force-based VIBF has been associated with improved goal-directed task performance in psychophysical experiments [11], as well as with virtual [12] and real prosthetic arms and hands [13-15]. Others have shown that increases in task accuracy with force-based VIBF are accompanied by a decrease in movement speed [16], or no differences in performance compared to controls [17].

In contrast to force information, the merits of providing information about prosthesis kinematics, i.e. position and velocity, has received less attention [18]. In a single-subject study Mann and Reimers [19] showed that signaling prosthetic limb position with continuous VIBF improved performance in a reaching task by as much as 50%. More recently, Bark et al. [20] had healthy subjects practice moving a force-controlled cursor along a line to different targets, and used VIBF and a skin-stretch manipulation to provide information about the cursor’s position. There appeared to be a performance benefit with VIBF, but Bark et al. note that this may have been due to practice effects and not the VIBF *per se*. Considering these reports, the usefulness of position-based VIBF for goal-directed tasks is unclear. Moreover, we are unaware of studies that have tested the value of velocity-based VIBF feedback. Velocity feedback for a prosthesis could provide “forward-looking” information (i.e. a rate of change suggests a future position), which may be beneficial for prosthesis control by offsetting visual processing delays [21]. When combined with visual feedback, velocity-based VIBF could provide a greater performance benefit compared to position-based VIBF. While velocity could be visually estimated from the moment-by-moment changes in position, this might add processing delays. Providing velocity information directly through VIBF could bypass this limitation. On the other hand, if nominally precise [22] vision-based positional information is absent, it may be of greater value to provide position-based VIBF, rather than having velocity-based VIBF as the sole source of feedback.

It is also unclear whether the effects of VIBF depend on the phase of application, i.e. during the acquisition of a skill or after proficiency has been obtained. To control a prosthetic arm, a user must learn the relationship between their inputs (e.g. muscle activity for a myoelectrically controlled prosthesis) and the resulting prosthesis motion. Augmenting visual feedback with kinematic VIBF may facilitate this process and speed skill acquisition. For example, Lieberman and Breazeal [23] asked healthy individuals to reproduce various arm movements while joint position VIBF was provided, and showed that VIBF increased the rate of skill acquisition compared to a no-feedback condition. However, movement speed was not explicitly considered in the analysis and there was some indication that the participants may have moved slower with VIBF, and therefore it is unclear whether VIBF increased skill. We define skill as a shift in the speed-accuracy relationship [24,25], i.e. when movements become faster without sacrificing accuracy, or more accurate without sacrificing speed. Alternatively, adding VIBF during skill acquisition could be a hindrance because the feedback is not “natural”. Therefore, the user might need additional time to learn how to map VIBF to prosthesis motion.

We clarified these issues by testing the effects of kinematic VIBF while healthy young adults learned to perform a goal-directed task with a virtual myoelectric prosthetic arm. During early skill acquisition we hypothesized that velocity-based VIBF would improve performance faster than visual feedback alone (and position-based VIBF) because velocity provides forward-looking information that can offset visual processing delays (Hypothesis 1; H1). We then determined whether velocity-based VIBF provides similar benefits later in skill acquisition. Again, with vision we expected velocity-based VIBF to be beneficial and not position-based VIBF due to lower spatial resolution compared to visual estimates of position (Hypothesis 2; H2). Without vision, we expected supplemental position-based VIBF to be most beneficial for performance (Hypothesis 3; H3).

## Methods

### Overview

Two experiments were performed. The first experiment tested the effects of kinematic VIBF on subjects’ ability to learn and perform a goal-directed task with a virtual myoelectric prosthetic arm. The second experiment tested the ability of subjects to discriminate between different virtual prosthetic arm motions using kinematic VIBF.

### Experiment 1 (Virtual arm task)

**Figure 1:**
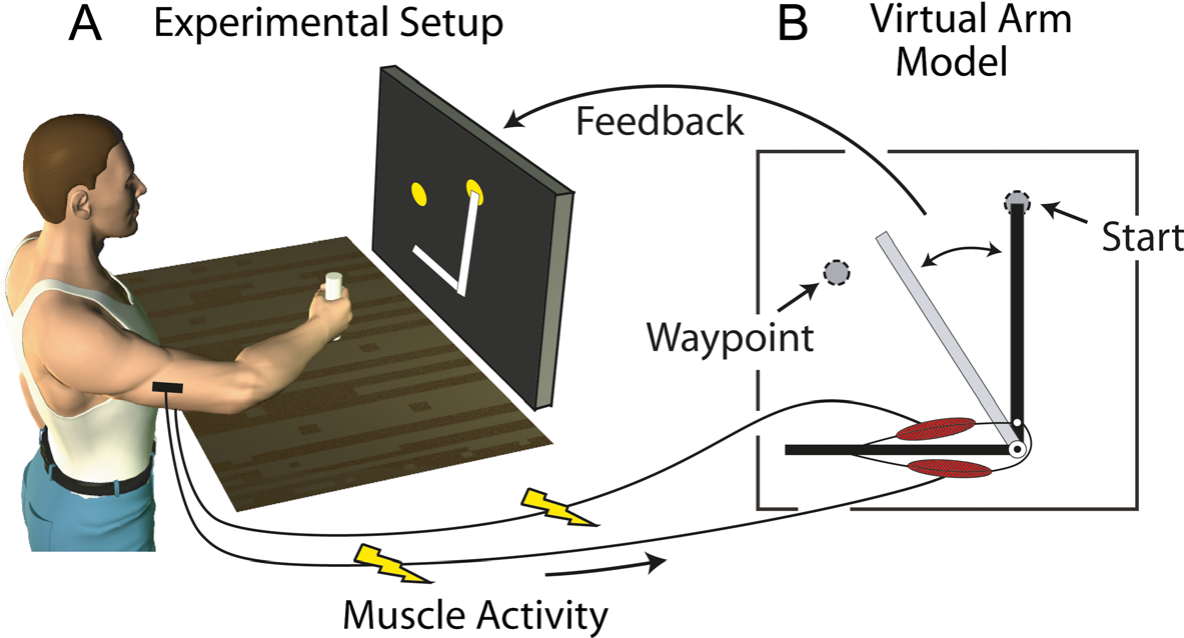
**Subjects performed a goal-directed task by controlling a virtual prosthetic arm with their muscle activity.** Biceps and triceps muscle activity was measured from subjects’ stationary dominant arms using electromyography **(A)**. The task was to move the virtual arm counterclockwise through a waypoint and then clockwise back to a circle identifying the starting position **(B)**. Subjects were instructed to move the virtual arm as quickly as possible and stop as close to the center of the starting circle as possible.

Twenty-seven healthy young adults performed a goal-directed task with a myoelectric virtual prosthetic arm (Figure 1). Subjects were randomly assigned to one of three groups, with nine subjects in each group. Two groups received amplitude modulated VIBF. In one group the vibration magnitude was based on the virtual arm’s position, and in the other it was based on the virtual arm’s velocity. A control group received no vibration. Subjects practiced the task for an initial adaptation phase of 240 trials. During this phase the VIBF groups received both visual and VIBF feedback; the control group only received visual feedback. This was followed by another 180 trials in which the feedback conditions were manipulated for the VIBF groups: visual feedback and VIBF were removed separately and at the same time (i.e. no sensory feedback of the virtual arm). Prior to participation, subjects read and signed an informed consent document approved by the Northeastern University Institutional Review Board.

### Musculoskeletal model

#### Virtual arm model

A virtual prosthetic arm was created in Matlab® (MathWorks®, Natick, MA).The virtual arm rotated about a hinge joint with a moment of inertia equal to 0.24 kgm^2^ [26]. A pair of antagonistic two-element Hill-type [27,28] muscle models produced forces to accelerate the arm. One muscle model represented the lumped behavior of the elbow flexors and a second modeled the lumped elbow extensors. Each muscle model had a maximal isometric strength (*P*_0_), a length-dependent strength defined by a force-length relation [29], a velocity-dependent strength defined by a force-velocity relation [27], and a series-elastic stiffness defined by a force-extension relation [30]. Similar muscle models have been used previously [31,32]. Parameters defining these relationships were adapted from the SIMM (MusculoGraphics Inc., Santa Rosa, CA) musculoskeletal modeling software [33]. The force-length relation was defined by an optimal contractile element length *L*_0_ (flexor = 0.132 m; extensor = 0.114 m) and a parabolic force-length relation with width coefficient *W* (54-146% *L*_0_). The force-velocity relation was defined by the normalized Hill coefficients *a/P*_0_ (0.25) and *b/L*_0_ (2.53 s^−1^), and the eccentric plateau *ε* (1.8 *P*_0_). The series-elastic stiffness was defined by a second-order polynomial with coefficients *α* (0.0258) and *β* (52.3) and the slack length *L*_S_ (flexor = 0.192 m; extensor = 0.098 m). Values for *α* and *β* were based on Bahler [30]. The lumped elbow flexor muscle model *P*_0_ was equal to the sum of the long and short head of the biceps brachii, brachialis, and brachioradialis (*P*_0_ = 2308 N; from SIMM). The lumped elbow extensor model *P*_0_ was equal to the sum of the short, long, and lateral heads of the triceps brachii (*P*_0_ = 2047 N; from SIMM). Virtual musculotendon lengths and moment arms vs. elbow joint angles were based on SIMM models. The lumped flexor and extensor length vs. angle relation was the average of the relations for the SIMM elbow flexor and extensor muscles, respectively. The individual muscle SIMM moment arms vs. elbow angle relations were averaged in a similar fashion to produce relations for the lumped flexor and extensor muscle models. An elastic torque *T*_*P*_ prevented the virtual limb from circling around the axis of rotation, such that *T*_*P*_ *= ab*^*(θ+c)*^*–ab*^*(−θ+c)*^, where a = 1.0 × 10–15, b = 0.65, and c = −10.6. A frictional torque *T*_*F*_ was added to mimic a limb rotating on a planar surface, based on the rotational friction model used in Matlab® Simscape™ (see [32] for details). This frictional torque also adds to the stability of the virtual arm model. A formal stability analysis of the model was not performed due to the significant nonlinearities present in the muscle dynamics. Qualitatively the virtual arm model was “well behaved”, i.e. it did not exhibit growing oscillations or rapid unintended accelerations in response to myoelectric inputs. Additional details related to the virtual arm model are provided in Hasson [32].

### Experimental setup

#### Apparatus

Participants sat in a chair and faced a computer monitor that displayed the virtual arm (Figure 1). Their right shoulder was flexed 75° (0° = anatomical position) and their elbow was flexed 45° (0° = full elbow extension). The lower arm rested on a horizontal support surface, and the subjects grasped a fixed bar with their right hand in a neutral position (all subjects were right-hand dominant). The bar allowed subjects to activate their elbow flexors and extensors via isometric contractions. Subjects’ arms remained in this fixed (isometric) position throughout the experiment. A fixed arm was used because in this state the electromyographic signal is a more direct measure of descending neural commands, compared to a freely moving arm in which neural commands are more heavily modified by intrinsic proprioceptive feedback.

#### Muscle activity measurement

Biceps and triceps muscle activity was monitored with a wireless electromyography system (Myon AG, Baar, Switzerland). The skin was shaved, rubbed with an abrasive gel, and cleaned with alcohol. Electrodes were positioned in a bipolar configuration in the center of the biceps and triceps (lateral head) muscle bellies, and were oriented parallel to the fibers with an inter-electrode distance of 2.0 cm. After placement, the electrodes were covered with elastic wrap. Motion artifacts were negligible due to the fixed arm posture and a 5 Hz high-pass filter applied by the electromyography system. Amplified muscle activity was rectified and filtered using a custom analog circuit that included a fifth-order low-pass Butterworth filter (MAX280; Maxim Integrated Products, Inc.; San Jose, CA, USA) with a cutoff frequency of 4 Hz [34].

#### Virtual interface

The filtered biceps and triceps muscle activity was sampled using an analog-to-digital converter (PCI-6289; National Instruments, Austin, TX) and these signals served as the neural commands to the virtual flexor and extensor muscle models. There was a fixed 16 ms delay between the transmission of the recorded muscle activity at the measurement sites and sampling by the computer. The signals were then subjected to first-order excitation-activation dynamics with activation/deactivation time-constants of 10 and 50 ms, respectively [35], and converted to forces via the lumped muscle models. These forces were multiplied by the muscle moment arms to produce torques about the virtual elbow axis of rotation (*T*_MUS_). The net joint torque *T*_NET_ was the sum of these active torques, the passive torque, and the friction torque (*T*_NET_ *= T*_MUS_ + *T*_P_ + *T*_F_). The virtual limb angular acceleration *α* was computed as *α* = *T*_NET_*/I*, where *I* is the moment of inertia of the virtual arm. A 4th-order Runge–Kutta algorithm [36] was used to integrate *α* to obtain virtual limb position and velocity. All computations and simulations were performed in Matlab®.

#### Vibrotactile setup

**Figure 2:**
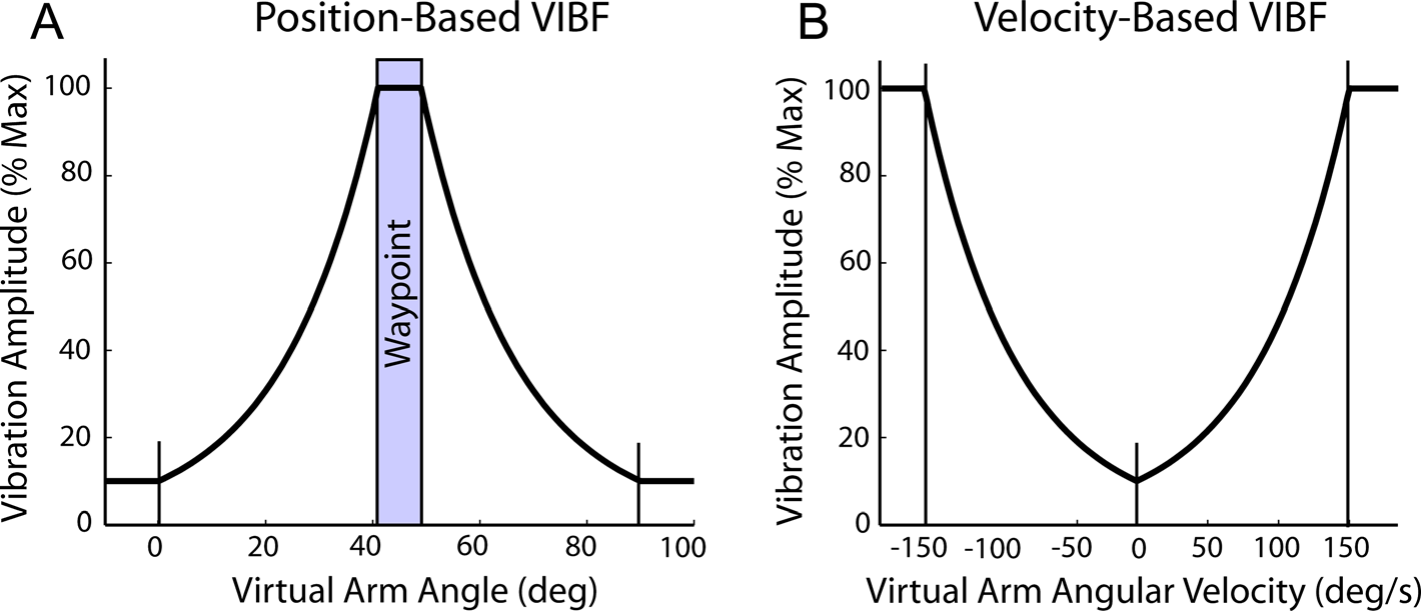
**The magnitude of the vibrotactile feedback (VIBF) was a function of either the virtual arm angle (A) or angular velocity (B) for position- and velocity-based VIBF, respectively.**

A linear resonant actuator (Model C10-100, Precision Microdrives, London, UK) was placed on the ulnar styloid of the dominant arm to provide VIBF during the task. A boney landmark was chosen to avoid placing the actuator directly over a tendon, which could vibrate the tendon and impose an unintended sensory illusion [37]. The device was applied to all subjects but did not vibrate for subjects in the control group. The actuator was controlled with a haptic driver (DRV2603, Texas Instruments, Dallas, Texas, USA) that ensured operation of the actuator at its resonant frequency (175 Hz) regardless of its mounting and other environmental features. The amplitude of the vibration was manipulated by adjusting the duty cycle of a pulse-width-modulated waveform produced by a signal generator (33210A, Agilent Technologies, Santa Clara, California, USA), which was controlled by the same Matlab® program that ran the virtual prosthetic arm interface. Amplitude modulation was used instead of frequency modulation as the former has shown to be more effective for sensory substitution, at least for the transmission of force information [38]. For the position-based VIBF group the vibration magnitude increased as the virtual arm moved from the starting position (0°) to the waypoint (45°). Bark et al. [20] used an exponential relation for signaling force information with VIBF due to evidence that amplitude perception is logarithmic [39]. Therefore, we used an exponential relation between the vibration magnitude and the virtual arm angular position (Figure 2A). If the virtual arm moved past the waypoint the vibration magnitude decreased again from the peak magnitude. Mapping the VIBF in this way makes VIBF saturate at the waypoint, creating a haptic landmark. We believed that incorporating this landmark would facilitate subjects’ task performance with the VIBF. For the velocity-based VIBF group the vibration magnitude increased exponentially with increasing virtual arm angular velocity until plateauing at 150°/s (Figure 2B). The plateau velocity was chosen after pilot data indicated that this was near the maximum angular velocity reached by participants. In both vibration conditions the lowest vibration magnitude was always 10% of the maximum. The vibration was disabled between trials. Subjects wore earplugs to mask the sound of the vibration.

### Experimental methodology

#### Virtual arm calibration

Subjects performed three maximal voluntary contractions (MVCs) with their arm in a fixed position. For the biceps, subjects tried to flex their elbow against the rigid experimental apparatus; for the triceps, they tried to extend their elbow. Subjects maintained each contraction at maximum for three seconds with 30 s rest between trials. The average maximum value of the hardware-filtered muscle activity voltages across the three trials was used for the calibration. This value represented 100% excitation to the virtual muscle models.

#### Task

After completing the calibration routine subjects performed the virtual prosthetic arm task. The virtual arm was drawn as a simple rotating line segment on the visual display (Figure 1B; note that the virtual muscles were not shown to the subjects). Subjects were instructed to use their muscle activity to perform a “slice” movement, which required the arm to be moved in two directions: counterclockwise to pass through a waypoint located 45° away and clockwise back to the starting location. Subjects were told to move the arm as quickly as possible and to stop the arm as close to the center of the starting location target circle as possible. For reinforcement the target circle turned from yellow to green when the angular error was within a success threshold of ±4° from the target center. When the virtual arm passed through the waypoint the waypoint disappeared. After each trial the movement time was displayed, defined as the time from when the limb left the starting circle to when the limb stopped moving (<4°/s for 0.3 s). For additional motivation, subjects’ fastest successful (stopped within ±4° of target) movement time was displayed, and if a given trial exceeded this time the program “applauded” and the fastest time was updated.

#### Experimental protocol

**Figure 3:**
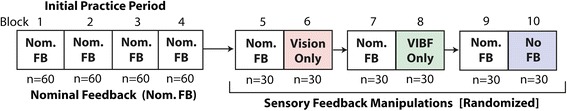
**Experimental protocol.** Subjects completed an initial practice period (Blocks 1–4) in which they practiced the task with their group-assigned nominal feedback condition: 1) only visual feedback (control group), 2) vision with position-based vibrotactile feedback (VIBF), and 3) vision with velocity-based VIBF. Next, the feedback conditions for the two VIBF groups were manipulated in random order (Blocks 5–10). The manipulations included Vision Only, VIBF Only, and No Feedback.

Subjects were asked to practice the virtual arm task for an initial adaptation phase consisting of four blocks of 60 trials (240 total) with nominal feedback depending on group assignment (Blocks 1–4, Figure 3). All groups received visual feedback of the virtual arm during this phase. This was done because we wanted to investigate the effects of VIBF during skill acquisition. Also, we found that learning to control the virtual arm without seeing the virtual arm move is extremely difficult (as demonstrated by pilot work), and most prosthesis users have visual feedback available during initial adaptation.

For the two VIBF groups, the initial adaptation period (Blocks 1–4, Figure 3) was followed by another six blocks of 30 trials in which the feedback conditions were manipulated in random order (Blocks 5–10, Figure 3). The latter blocks occurred in pairs. In the first block of 30 trials (after the initial adaptation period) subjects experienced the nominal feedback condition (vision plus VIBF), and this was immediately followed by a second block of 30 trials in which the feedback manipulation was applied. These manipulations included: visual feedback removed leaving only vibration (Vibration Only), vibration turned off leaving just visual feedback (Vision Only), or both visual feedback and vibration removed (No Feedback). For the Vibration Only condition, the virtual arm disappeared once it started moving, and reappeared at the end of the trial so subjects could see the result of their actions. The waypoint was shown but it did not disappear when the (hidden) virtual arm passed through as this would provide position and/or velocity information to the subjects. In this case, after the trial ended the data collection program indicated whether the waypoint was passed. The control group also completed Blocks 5–10, but without any sensory manipulations, i.e. with only visual feedback. Before each trial there was a 2 s pre-trial preparation time and post-trial result feedback was presented for 2 s. Each trial took 1–2 s, depending on the movement time. Each block of 60 trials took between 5–6 min with 2 min rest breaks between each block. The entire virtual arm practice session lasted 70–80 min.

### Data analysis and reduction

#### Dependent measures

The three dependent measures were: 1) movement time, 2) absolute angular error, and 3) skill. Movement time was the time from when the limb left the starting circle to when it came to rest. The absolute angular error was the absolute value of the angular difference between the virtual limb’s final position and the center of the starting circle. The skill measure is a composite of movement time and error since subjects could trade-off either quantity, i.e. they could move slower and be more accurate, or vice-versa. The skill measure is similar in concept to that used by Reis et al. [40], i.e. skill is low for slow and inaccurate movements, and high for fast and accurate movements. To correct for differing units of measure, movement time and error were scaled to a range covering 95% of the data across practice (error range: 0-13°; movement time range: 0.8-2.3 s). When plotted against each other, the two scaled measures give a single point in Cartesian space and the distance to this point is a representation of skill (see Hasson [32]). This distance was multiplied by −1 so an increase represented improved skill, and the measure was offset by shifting the measure by an amount equal to the average skill value for the first 5 trials of the control subjects (this skill level became zero-skill). Trials in which the virtual arm did not pass through the waypoint were excluded from analysis.

#### Analysis

The effects of VIBF on the rate of skill acquisition were assessed by fitting exponentials to each subject’s binned error, movement time, and skill data using a nonlinear least squares method (bin size = 10 trials). These fits included the initial practice period (Blocks 1–4), plus the three 30-trial nominal feedback practice blocks during the sensory manipulations (Blocks 5, 7, and 9). This uses the maximum amount of available data for the exponential fits. For Blocks 7 and 9 the first five trials were discarded to minimize the influence of washout effects from prior sensory manipulations. The exponential time constants reflected the rates of change in error, movement time, and skill. As a complement to this analysis, the average error, movement time, and skill were computed for the first 10 trials of Block 1, capturing very early practice, and the last 55 trials of the initial practice period (Block 4), and these two measures were subtracted to obtain a measure of the change across practice (late minus early). Block 1 was compared with Block 4 (Figure 3) because analysis showed no differences between the performance on Block 4 and each of the subsequent nominal feedback blocks (i.e. Blocks 5, 7, and 9; tested with a two-way ANOVA with group and block as between- and within-subjects factors, respectively; there was no main effect of block number: *p* > .05). For each subject the average and standard deviation of the maximum angular excursion of the virtual arm (i.e. the turn-around point) was computed to help characterize subject movement strategies. How performance changed in response to manipulations of visual and VIBF feedback (H2 & H3) in the groups who trained with VIBF was assessed by computing the average error, movement time, and skill for each of the 30-trial blocks with the sensory manipulations (last 25 trials of Blocks 6, 8, and 10).

### Statistics

To test our hypothesis about early skill acquisition (H1), a between-subjects analysis was performed using one-way ANOVAs with group as the independent variable (control, position-based VIBF, or velocity-based VIBF). Dependent variables were the exponential time constants and the change from early to late practice for error, movement time, and skill. Additionally, one-sample t-tests were performed separately for each group to determine if the change in performance was significant (i.e. did a given group improve their performance?). Two additional sets of statistical analyses were performed on the sensory manipulation data. These analyses used the change in performance (error, movement time, and skill) across the manipulation, computed as the performance with a manipulation (Vision Only, VIBF Only, or No Feedback) relative to performance on the nominal feedback trials immediately preceding the manipulation. To analyze subject responses to VIBF removal in the Vision Only condition (H2), a one-way ANOVA was performed to test for between-group differences, and one-sample *t*-tests were performed separately for each group to test whether VIBF removal elicited a significant change in performance across the manipulation. To determine whether there were differences in subject responses to removal of visual feedback vs. removal of all feedback (H3), a two-way ANOVA was performed with group as a between-subjects factor and condition (VIBF Only vs. No Feedback) as a within-subjects factor. When appropriate, post-hoc comparisons were performed using Tukey’s honestly significant difference. Significance was set at *p* < .05 for all tests.

### Experiment 2 (Vibrotactile feedback discrimination)

**Figure 4:**
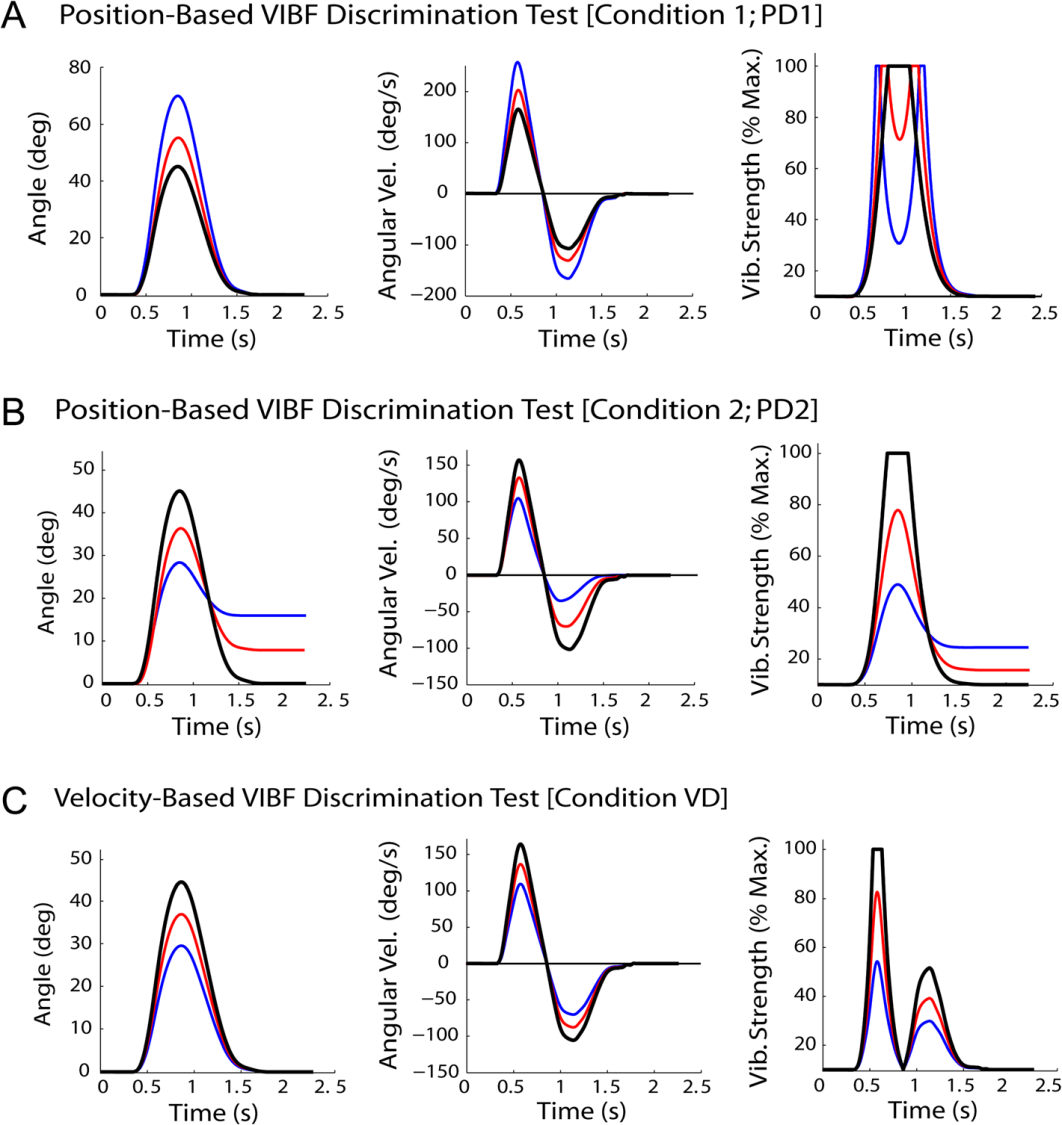
**Virtual arm angular position and velocity profiles and magnitude of vibrotactile feedback (VIBF) for each of three VIBF discrimination conditions.** In a position‐based VIBF discrimination condition, the maximum excursion of the virtual arm was varied **(A)**. In a second position‐based VIBF discrimination condition, both the maximum excursion and stopping location of the virtual arm were varied **(B)**. Finally, in a velocity‐based VIBF discrimination condition, the maximum virtual arm angular velocity was varied **(C)**. Kinematic profiles for the standard (black), the initial comparison (blue), and one intermediate comparison (red) are shown (see text for details).

A second experiment with a new group of five young healthy adults was performed to assess the ability of participants to discriminate between different position- and velocity-based VIBF profiles. A two-interval forced-choice sensory discrimination paradigm was used [41], which required subjects to make repeated discriminations between pairs of standard and comparison VIBF profiles. There were three VIBF discrimination conditions. Each condition used the same standard virtual arm VIBF profile, but had different comparison VIBF profiles. The standard was created by selecting a representative trial from *Experiment 1* that had a maximum angular excursion of 45° (just touching the waypoint) and stopped within 1° of the goal with no discernible corrective actions (Figure 4; black lines).

For the first test condition, Position Discrimination 1 (PD1), the angular distance that the virtual arm moved past waypoint was varied. The comparison profile was created by multiplying the standard profile by 1.55, which caused the maximum angular excursion to increase to 70° (Figure 4A; blue lines). This “stretched” the displacement profile; however, the virtual arm still came to rest back at the starting position. For the second test condition, Position Discrimination 2 (PD2), the stopping location of the virtual arm was also varied as follows: 1) After computing the angular velocity *ω* for each data point *i* from the angular displacement data using numerical differentiation, a new angular velocity array $\widehat{\omega}$ was determined as ${\widehat{\omega}}_i={\omega}_i\left(1-\mathrm{z}\right)$, where *z* is a vector of linearly spaced numbers that increased with the sample number *i* from 0 to a maximum of 1.4. This gradually reduced the virtual arm velocity (relative to the standard) as time progressed. 2) Integration of $\widehat{\omega}$ gave a displacement profile in which the virtual arm only moved 28° towards the waypoint and stopped 16° short of the target (Figure 4B; blue lines). Note that in PD1 the comparison position-based VIBF profile had two peaks because the virtual arm moved past the waypoint, but the PD2 comparison VIBF profile had a single peak. To test velocity-based VIBF discrimination, a third condition, Velocity Discrimination (VD), was created by multiplying the standard angular position profile by 0.67, which reduced the peak velocity from 165°/s to 110°/s and reduced the maximal angular excursion to 30° (Figure 4C; blue lines). For all three test conditions, the total virtual arm movement time, and the time spent in each movement direction remained the same, so subjects could not use differences in the overall vibration duration for discrimination.

For each of the three conditions, a familiarization session was performed followed by discrimination tests. The condition order was randomized but a block of discrimination tests always followed a familiarization session. For each familiarization session subjects were presented with 20 virtual arm movements using random manipulations of the standard virtual arm motion and VIBF profile. The type of manipulation was consistent with the upcoming discrimination test condition, i.e. for PD1 and VD the standard profile was “stretched” and “shrunk” by different amounts, respectively, and for PD2 different weighting factors were used. The moving virtual arm was visible to subjects during these trials in addition to VIBF. This allowed the naive subjects to understand how the VIBF related to the virtual arm movement. Each familiarization session took about two minutes.

After each familiarization session, subjects performed one of the three discrimination conditions (PD1, PD2, or VD), in which visual feedback was removed and only VIBF was provided. For the first discrimination test the standard and initial comparison VIBF profiles were presented in random order. This was followed by a prompt asking subjects to choose whether the first or second trial (i.e. the standard or the comparison) was associated with a motion that either: moved farther past the waypoint (PD1), did not reach the waypoint and stopped short of the goal (PD2), or moved slower (VD). Subjects used their uninvolved hand to press either the “1” or “2” key to indicate their choice. A “correct” or “incorrect” was displayed on the screen depending on the answer. Following this a new pair or standard and comparison VIBF profiles was presented.

For PD1, following every three non-consecutive correct responses, the angular excursion of the comparison VIBF profile was reduced by 0.8 dB (for the initial comparison, 70° vs. 45°, the ratio is 1.56 = 0.8 dB), becoming closer to the standard. Once the comparison maximum excursion became less than 57.5° the step-size was halved (0.4 dB). For every incorrect response the comparison profile maximum angular excursion was increased in the same way. For PD2 the virtual arm angle at movement termination was decreased 0.8 dB after three correct responses, stopping closer to where the standard stopped. After an incorrect response the stopping angle was increased (note that 45° was added to the standard and comparison termination angles before computing the ratio to make the size of the changes consistent with PD1; the step size was halved in the same way). Finally, for VD the peak velocity was increased or decreased by 0.8 dB after three correct or one incorrect response(s), respectively. In each condition subjects were presented with 50 trials and asked to make a total of 25 comparisons, which took about 15 minutes to complete. This struck a balance between the number of data points acquired and subject fatigue.

## Results

### Experiment 1 (Virtual arm task)

**Figure 5:**
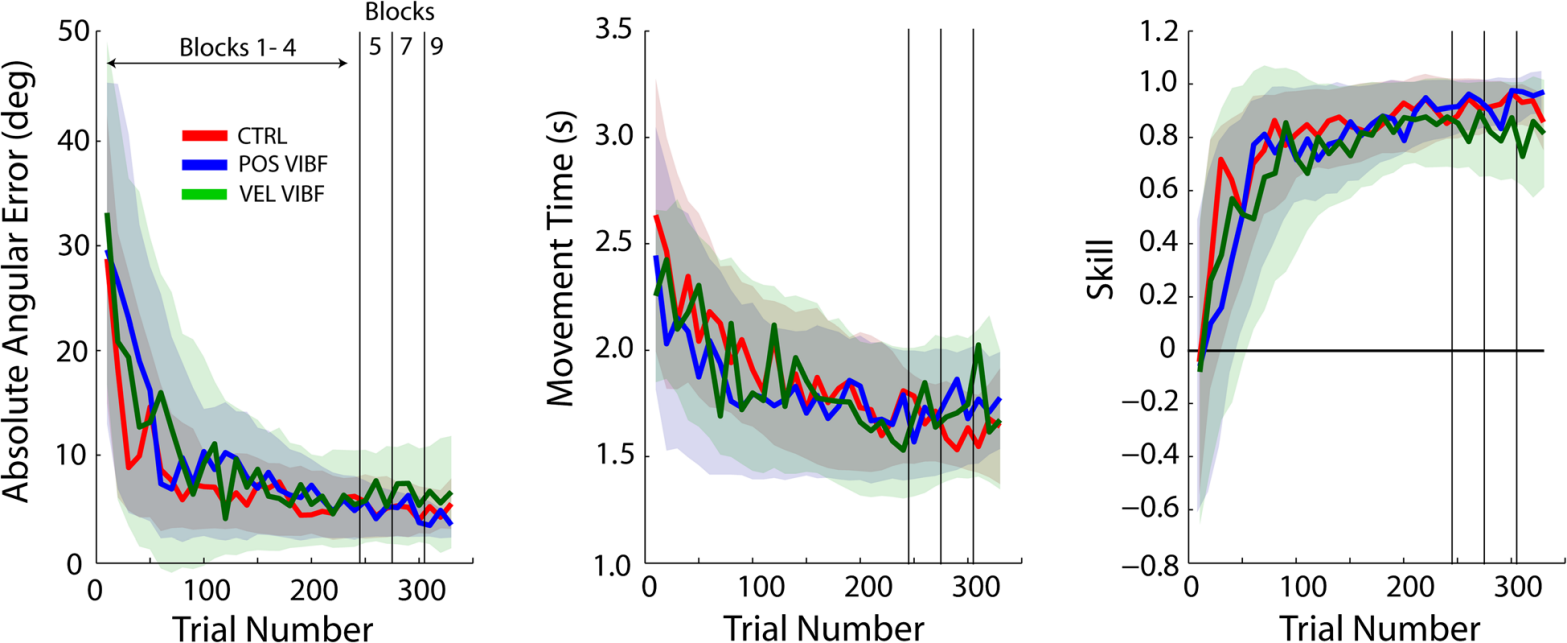
**With practice all groups decreased their end-point error, increased their movement time, and consequently increased their skill at performing the virtual arm task.** However, there were no group differences in the rate of improvement in error (left panel), movement time (middle panel) or skill (a composite of accuracy and speed; right panel). The control group (CRTL) was provided with only visual feedback during practice. The other groups received vibrotactile feedback (VIBF) that was proportional to either the virtual arm angular position (POS VIBF) or angular velocity (VEL VIBF). The shaded areas show the smoothed between-subjects standard deviations within each group across practice. For the VIBF groups the last three blocks (5; 7; 9) had sensory manipulations intermixed, but these data are not shown here for clarity.

**Figure 6:**
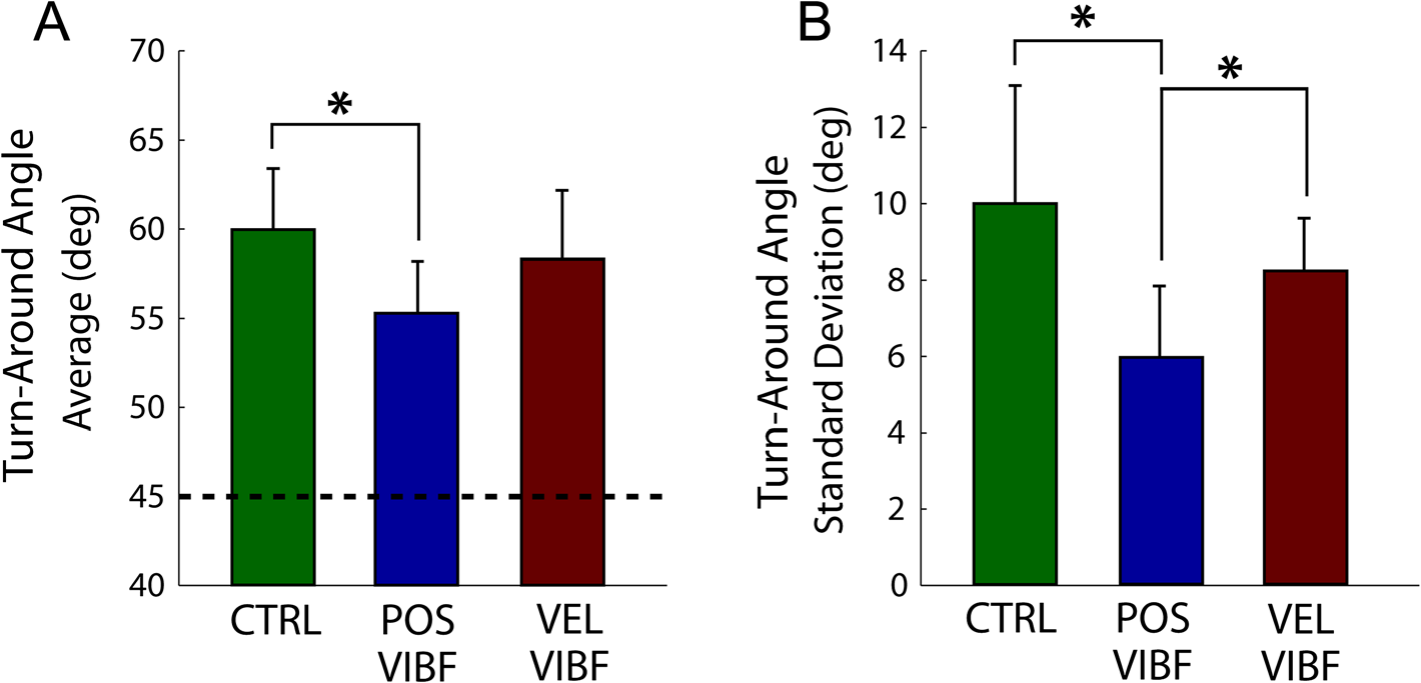
**Maximum turn-around virtual arm angle after it passed the waypoint.** A larger angle indicates that the arm moved further past the waypoint. Both the group mean **(A)** and average standard deviation across trials **(B)** are shown. Data are from late-practice (Block 4). The waypoint was at 45°, indicated by a dashed line in A. *Significant differences between groups.

Within the practice period subjects were able to improve their control of the virtual myoelectric prosthetic arm. The control group and both kinematic VIBF groups demonstrated a significant reduction in error (*p* < .001) and movement time (*p* < .001) and an increase in skill (*p* < .001) from early to late practice (Table 1; Figure 5). Analysis of the group data showed that in late practice (Block 4) the control group moved the virtual arm farther past the waypoint (Figure 6A), and had more variability in this angular excursion over repeated trials (Figure 6B) compared to the position-based VIBF group (*p* = .006 for mean excursion; *p* = .001 for standard deviation) but not the velocity-based group (*p* = .285 for mean; *p* = .102 for standard deviation). There was no difference between the position- and velocity-based VIBF groups for the maximum virtual arm angular excursion (*p* = .071) but there was for the variability of the angular excursion (*p* = .047), with position-based VIBF less variable than velocity-based VIBF. In late practice (last 55 trials) the percentage of trials that passed through the waypoint was 96 ± 3%, 93 ± 10%, 96 ± 7% for the control, position-based VIBF, and velocity-based VIBF groups, respectively (mean ± standard deviation).

**Table 1:** **Performance changes in nominal feedback conditions (mean ± between-subjects standard deviation)** ^**a**^

**Measure**			**Group**			***p*** **-value** ^**b**^
			**Control**	**Position VIBF**	**Velocity VIBF**	
Data	Early practice	Error (deg)	28 ± 31	31 ± 13	32 ± 16	.873
		Movement time (s)	2.6 ± 0.6	2.3 ± 0.7	2.2 ± 0.5	.324
		Skill	0.0 ± 1.0	−0.09 ± 0.5	−0.1 ± 0.6	.924
	Late practice	Error (deg)	6.3 ± 5.6	5.6 ± 2.4	7.3 ± 6.9	.775
		Movement time (s)	1.8 ± 0.3	1.7 ± 0.3	1.6 ± 0.3	.396
		Skill	0.9 ± 0.2	0.9 ± 0.07	0.8 ± 0.2	.672
	Improvement	Error (deg)	22 ± 25	25 ± 16	24 ± 14	.859
		Movement time (s)	0.8 ± 0.8	0.6 ± 0.6	0.6 ± 0.5	.710
		Skill	0.9 ± 0.6	1.0 ± 0.5	0.9 ± 0.5	.791
Exponential fit	Improvement rate^c^	Error	27 ± 19	40 ± 32	34 ± 36	.723
		Movement time	44 ± 22	35 ± 34	59 ± 24	.327
		Skill	40 ± 56	36 ± 28	46 ± 59	.933
	Plateau	Error (deg)	5.5 ± 1.4	5.9 ± 1.8	4.5 ± 2.7	.718
		Movement time (s)	1.6 ± 0.2	1.7 ± 0.3	1.4 ± 0.6	.748
		Skill	0.90 ± 0.04	0.94 ± 0.13	0.78 ± 0.3	.350

^a^Nominal conditions are: 1) only visual feedback (the control group), 2) vision with position-based vibrotactile feedback (VIBF), and 3) vision with velocity-based VIBF.

^b^For main effect of group.

^c^Units are trials taken for measure to improve by about 63.2%.

We expected that subjects who received velocity-based VIBF to augment visual feedback, and not position-based VIBF, would have faster improvements in performance compared to subjects who only had visual feedback available (H1). This was based on the conjecture that velocity provides forward-looking information to offset visual processing delays, and because vision-based positional information has a relatively high resolution. However, there were no differences in the rate of improvement in error, movement time, and skill (composite of accuracy and speed) between the control, position-based VIBF, and velocity-based VIBF groups (Table 1; Figure 5). Further, providing velocity information via VIBF did not provide any advantage over positional VIBF information. These results are supported by the analysis of the change in error, movement time, and skill across practice (late minus early practice), which also showed no group differences (Table 1).

**Figure 7:**
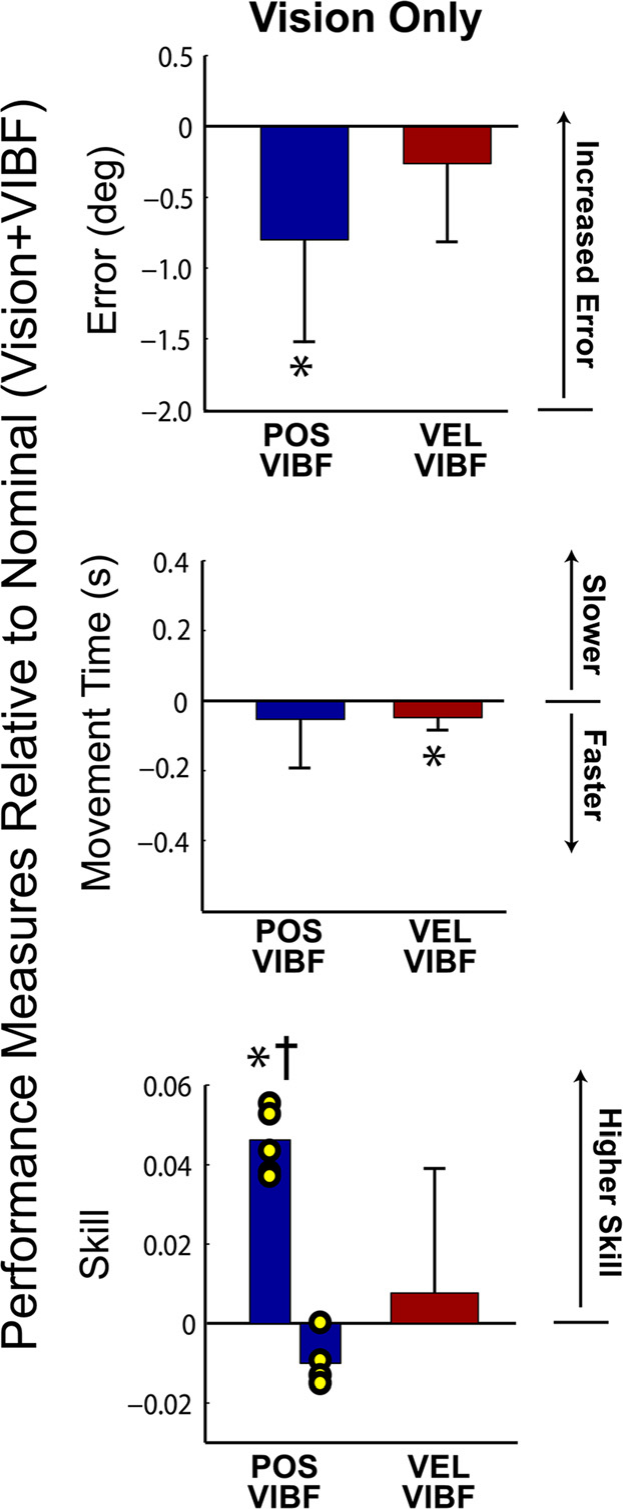
**Effects of removing vibrotactile feedback (VIBF) on measures of task performance (error, movement time, and skill) for groups receiving position-based (POS VIBF) and velocity-based (VEL VIBF) vibrotactile feedback.** All measures are relative to performance on the nominal feedback condition, in which visual feedback was available in addition to VIBF. A subset of participants in the POS VIBF group improved their skill when VIBF was removed leaving only vision (†), while other participants showed no change in skill (individual subject data points are overlaid). Means and standard deviations shown. *Significantly different from zero at p < .05.

We next focused on whether kinematic VIBF benefits performance after a measure of task proficiency had been obtained. It was anticipated that participants who trained with velocity-based VIBF would show a decrement in skill with VIBF removal, but those who trained with position-based VIBF would show no decrement (H2). For velocity-based VIBF, removal of VIBF (Vision Only condition) was associated with no change in error (*p* = .222; Figure 7, top), a small *decrease* in movement time (*p* = .008; Figure 7, middle), and no change in skill (*p* = .515; Figure 7, bottom). When VIBF was removed in the position-based VIBF group the error became *smaller* (*p* = .010) with no change in movement time (*p* = .287). There was a bimodal distribution for the skill measure in the position-based VIBF group; some subjects decreased error and movement time (increasing skill), but others traded-off speed for accuracy, i.e. movement time increased and error decreased (no net change in skill). Therefore, the two Vision Only subgroupings were analyzed separately. One subgroup (n = 4) had no change in skill when VIBF was removed (*p* = .063), the other subgroup (n = 5) had an *increase* in skill (*p* < .001; Figure 7). There were no differences between the position- and velocity-based VIBF groups for the change in error and movement time due to VIBF removal in Vision Only (*p* > .600 for all comparisons). However, for Vision Only the position-based VIBF subgroup who improved their skill with VIBF removal performed better than the velocity-based VIBF group (*p* = .009), while the other position-based VIBF subgroup was not different from the velocity-based VIBF group (*p* = .165).

**Figure 8:**
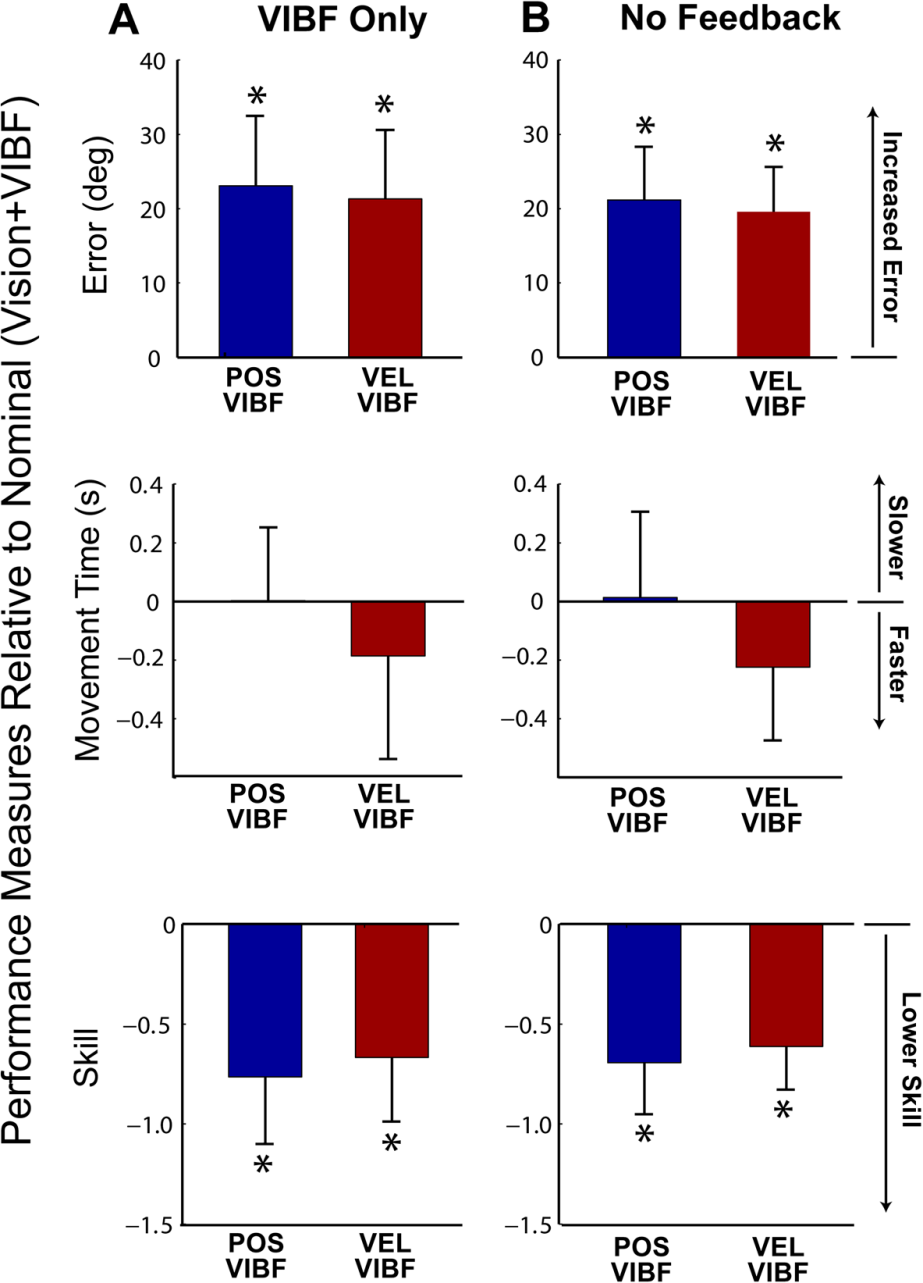
**Effects of removing vision (Vibrotactile Feedback [VIBF] Only; A) and removing both vision and VIBF (No Feedback; B) on measures of task performance (error, movement time, and skill) for groups receiving position-based (POS VIBF) and velocity-based (VEL VIBF) vibrotactile feedback.** All measures are relative to performance on the nominal feedback condition, in which visual feedback was available in addition to VIBF. Means and standard deviations shown. *Significantly different from zero at p < .05.

Finally, we posited that without visual feedback, task performance with kinematic VIBF would be better than with no feedback at all, and without visual feedback position-based VIBF would be associated with better performance than velocity-based VIBF (H3). To test this hypothesis, subject performance changes due to removal of visual feedback (VIBF Only; Figure 8A) were compared with those due to removal of all feedback (No Feedback; Figure 8B). Contrary to expectations, error and skill for VIBF Only was not different from No Feedback (no main effects; error *p* = .424; skill *p* = .437). This was true for both position-and velocity-based VIBF groups (i.e., no interaction between group and condition; error *p* = .854; skill *p* = .757). For movement time there was no effect of condition (main effect; *p* = .764), but the velocity-based VIBF group moved faster than the position-based group (*p* = .046), and this was consistent across the conditions (i.e. no interaction; *p* = .318).

### Experiment 2 (Vibrotactile feedback discrimination)

**Figure 9:**
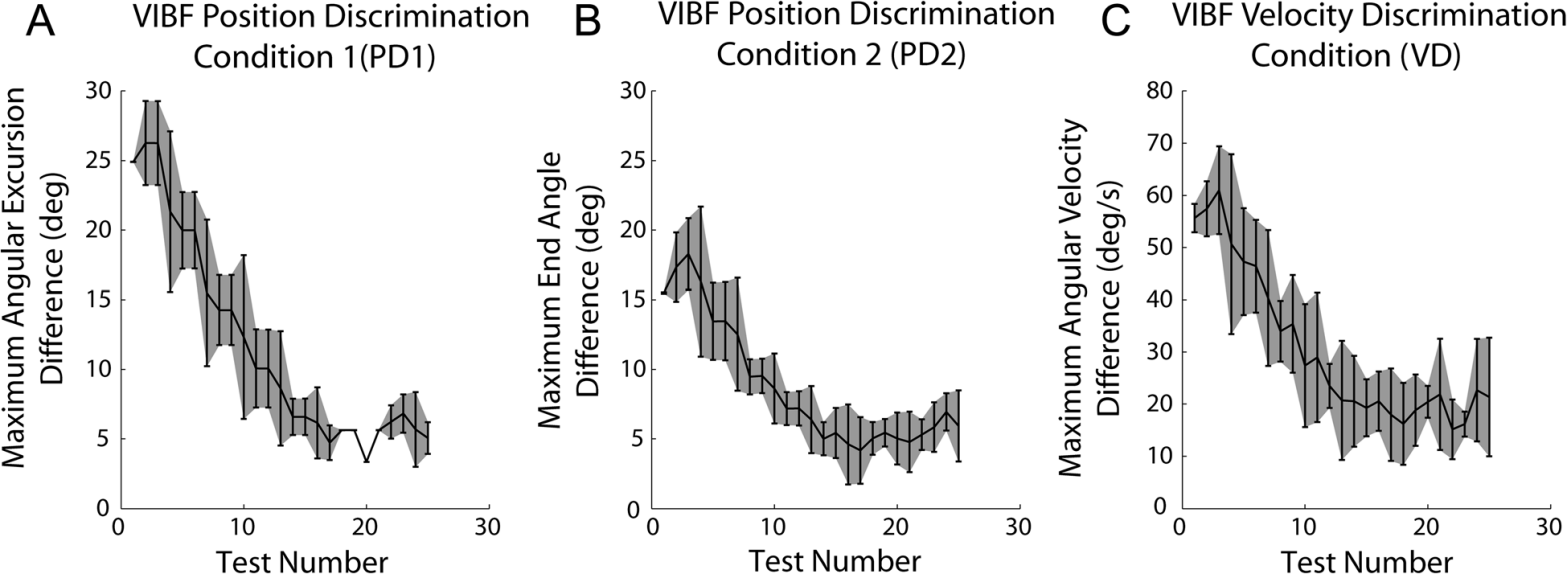
**Results of vibrotactile feedback (VIBF) discrimination tests showing the ability of subjects to discriminate between the maximum angular virtual arm excursion (A), the stopping location of the virtual arm (B), and the maximum virtual arm angular velocity (C) on the basis of VIBF alone (without visual feedback).** Means and between-subjects standard deviations are shown.

The purpose of this experiment was to assess how well subjects could discriminate between different virtual arm motions on the basis of VIBF alone. The results are summarized in Figure 9. For PD1 the maximum excursion of the virtual arm back-and-forth motion was manipulated. Using only VIBF, subjects were able to reliably detect a difference in the maximum angular virtual arm excursion of ~5° (Figure 9A). The results were similar for PD2; subjects could detect a difference in the stopping location of the virtual arm of ~5° (Figure 9B). Finally, for VD subjects could detect a difference in the maximum angular velocity of the virtual arm of ~20°/s (Figure 9C).

## Discussion

The purpose of this study was to investigate the effects of kinematic VIBF on learning to control a virtual myoelectric prosthetic arm. A control group of subjects learned to perform a goal-directed task with only visual feedback of the virtual arm, another group received an additional VIBF stimulus that depended on the virtual arm’s angular position, and a third group received an additional VIBF stimulus that was angular velocity-dependent. The main results were that neither position- nor velocity-based VIBF increased the rate of skill acquisition, and neither type of VIBF improved task performance; in some cases VIBF reduced performance. VIBF sensory discrimination tests showed that subjects could detect virtual arm angular position and velocity differences of about 5° and 20°/s, respectively, based on VIBF alone.

### Skill acquisition

Adding both position- and velocity-based VIBF to visual feedback failed to increase subjects’ rate of improvement in error, movement time, and skill relative to controls that practiced with only visual feedback. We did not expect position-based VIBF to have a significant effect on skill acquisition because visual feedback already provides accurate positional information [22]. However, we hypothesized that adding supplemental velocity-based VIBF would increase the rate of performance improvement in the virtual arm task. Our rationale was twofold. First, velocity-based VIBF provides information about how quickly the position of the virtual arm is changing, which may offset delays involved in visual information processing [21]. Second, we posited that velocity-based VIBF might assist participants in developing a map between how they activate their muscles and how the virtual arm responds. However, the data do not support these expectations.

One explanation for the lack of hypothesis support could be that the addition of kinematic VIBF created an extra learning challenge for subjects. Not only did subjects need to learn the mapping between their muscle activations and the movement of the virtual arm, they may have also needed to learn the mapping between the magnitude of the vibration and the virtual arm movement. However, the lack of group differences during the initial skill acquisition phase suggests that this was not the case. Future work could investigate this further by having subjects first practice the task with only visual feedback (for an extended time) and then adding VIBF. A subsequent performance dip may reflect the challenge posed by integration of VIBF information into subject control strategies. It is also possible that the central nervous system could have chosen to ignore the VIBF, which is consistent with the suggestion that humans give less weight to sensory signals that require more challenging coordinate transformations [42]. However, it is unclear whether the subjects completely ignored the VIBF: the position-based VIBF group had less variability in the peak excursion of the virtual arm (i.e. the turn-around point) compared to the other groups, which could suggest that the subjects were sensitive to the haptic “landmark” provided by the VIBF relative to the waypoint. A final possibility is that for some subjects the VIBF was perceived as disturbing [13]. Indeed, after the experiment two participants reported that the VIBF was “annoying”, but the others did not.

### Late skill acquisition

The second objective of this study was to determine whether kinematic VIBF could elicit improvements in performance after some degree of skill had been obtained. Specifically, we hypothesized that after training with visual feedback and supplemental velocity-based VIBF, removal of VIBF would decrease performance, suggesting that velocity-based VIBF was beneficial. However, the results showed that when velocity-based VIBF was removed leaving only visual feedback, there was no effect on task performance.

Although we did not expect removal of position-based VIBF to have a significant effect with visual feedback present, its removal was associated with a *decrease* in error, showing that position-based VIBF had a *deleterious* effect on task performance. For the composite skill measure, a subset of subjects in the position-based VIBF group improved their skill with VIBF removal, while the others showed no change. These two sub-groupings were very distinct. The differential effects of VIBF on skill suggest that for some subjects, position-based VIBF may have interfered with their ability to perform the task. This is consistent with reports that some users find VIBF distracting, while other users are not bothered [43,44]. This result could also indicate that the presence of position-based VIBF interfered with subjects’ processing of vision-based positional information. Human visual angular discrimination thresholds are about 1.0 - 1.4° [22], while the sensory discrimination tests showed a roughly 5° discrimination threshold for VIBF. Thus, subjects may have been better served ignoring position-based VIBF when accurate visual information was available.

We also expected that VIBF would improve performance in the absence of visual feedback, as others have shown that proprioceptive information has the greatest effect when vision is absent [45]. Surprisingly, performing the task with only VIBF (and no visual feedback) was no different from performing with no feedback at all. This result was consistent for both position- and velocity-based VIBF groups. This further supports the null hypothesis that the type of kinematic VIBF provided in this study is not beneficial for controlling a virtual myoelectric prosthetic arm – even when VIBF was the only source of sensory feedback about the virtual arm’s motion.

Although the sensory discrimination tests showed that subjects could obtain kinematic information through position- and velocity-based VIBF, they could focus entirely on the VIBF during these tests. In *Experiment 1* subjects had to also issue motor commands for virtual arm control, and could have experienced difficulty using VIBF to update the relevant inverse and forward internal models [46], i.e. mapping VIBF to and from motor commands. This could be because the VIBF only stimulated cutaneous afferents near the skin surface and not deep proprioceptive structures, as the device was placed on a bone (ulnar styloid) and not directly over a muscle or tendon. Although VIBF detection thresholds within the glabrous skin of the fingertips are lower than for more proximal arm locations [47], the fingertips were not used as they would be unavailable for many prosthesis users (in the affected arm). The sensory conditions at the ulnar styloid may be more representative of other more proximal locations on the body, which could receive VIBF in an amputee controlling a prosthetic arm.

### Comparison with existing studies

Our study showed that kinematic VIBF did not facilitate performance, and in some cases hindered performance. In contrast, Mann and Reimers [19] demonstrated that continuous position-based VIBF improved the control of a prosthetic arm in a goal-directed reaching task, and Lieberman and Breazeal [23] reported that providing joint position VIBF during an arm movement matching task increased accuracy compared to a no-feedback condition. Differences could be because the Mann and Reimers study involved an amputee with a prosthetic arm (a single subject), instead of healthy adults controlling a virtual prosthetic arm. Lieberman and Breazeal had participants control their own arms instead of a prosthesis, and used eight vibrotactile devices that activated sequentially to indicate joint motion instead of a single amplitude-modulated VIBF device. On the other hand, our results are consistent with those of Bark et al. [20], who had healthy individuals control a virtual object and showed no clear performance benefit with VIBF (after accounting for practice effects). Because this literature focused on accuracy as the measure of performance, it is difficult to know with certainty how VIBF affected skill because changes in speed have an inverse effect on accuracy [25]. While Bark et al. reported no change in the average cursor velocity with VIBF, no statistical analysis was performed on this measure. Others have shown that providing force-based VIBF increases accuracy but decreases speed [16]. We measured both end-point accuracy and speed (i.e. movement time) because we did not constrain either of these quantities (participants were instructed to maximize both). Allowing subject’s room to co-vary accuracy and speed as they learn a task more closely mimics natural learning scenarios.

### Considerations and limitations

Participants received sensory information from their intact arm, in addition to that provided by the VIBF stimulus. This could pose a conflict because the kinematic information conveyed by intact sensors (e.g. muscle spindles) is different from that transmitted by the VIBF device. The former indicates the position and/or velocity of the actual arm, while the latter is of the virtual arm. This raises the question: which signals should the nervous system use to control the virtual prosthetic arm? The minimum intervention principle [48] states that corrections should be made only for errors that affect the task goal. Between the actual and virtual limbs, only the error information conveyed by the VIBF (and visual estimates of the virtual arm motion) was pertinent to the task goal, and therefore should be acted upon.

There were aspects of the vibration manipulations that may not have been optimal. There were differences in how the position- and velocity-based VIBF was mapped to vibration amplitude. The relationships between the virtual arm position/velocity and the magnitude of the vibration were exponential, which followed Bark et al. [20], who used exponential vibration amplitude mappings due to evidence the human amplitude perception is nonlinear [39]. For position-based VIBF the peak vibration was mapped to a known position. While in very early practice subjects often did not complete the full out-and-back slice movement due to their lack of skill, after the first block of practice trials most subjects moved through similar ranges of virtual arm motion. However, for velocity-based VIBF, the peak vibration strength was mapped to an arbitrary velocity reference. A subject with more skill can move the virtual arm with a higher velocity, and would therefore receive higher-magnitude vibration. This may make the amount of vibration experienced by subjects in the velocity-based VIBF group more variable than the position-based VIBF group. The location of the vibration device on the body could influence VIBF perception. However, Stepp and Matsuoka [49] tested a variety of stimulation sites for force-based VIBF and found similar improvements in object manipulation performance. However, further research is needed to see if this holds for kinematic VIBF information.

The results suggest that kinematic VIBF is not beneficial for learning to control a virtual myoelectric prosthetic arm. However, more study is needed before definitive conclusions can be made. Differences could emerge for tasks not investigated. Proprioceptive feedback could have a different role in tasks involving multiple joint motions compared to just one [50], and in tasks that involve more on-line corrections, e.g. with multiple waypoints. There are also a number of other variables that may influence the results, which were not explored. This includes the strength of the vibration and the relationship between the vibration strength and virtual arm position and velocity. For example, the vibration strength could reach maximum at the end of the arm range of motion instead of at a waypoint, position and velocity information could be combined, and a vibration device could be placed over both the biceps and triceps to provide signed VIBF. Instead of continuously modulating VIBF, discrete bursts of vibration could be provided; event-based VIBF has recently been shown to improve the control of a robotic hand [51].

It is also unclear how our results would extend to the use of actual physical prosthetic arms. In addition to differences associated with controlling a physical device, such as the presence of proprioceptive feedback from limb-prosthesis interaction forces, different control schemes could be used. While some prostheses respond to muscle activity in a proportional way, many use algorithms to classify muscle activity patterns, which then trigger a pre-programmed motion (see [52] for a review). In addition, our virtual arm model contained muscle models, which translated the muscle activity into forces that moved the virtual arm. Such models are not normally incorporated into prosthesis control; however, they may prove useful for future prosthesis designs (e.g. [53]).

## Conclusions

This study investigated the effects of providing kinematic VIBF on human control of a virtual myoelectric prosthetic arm. The results demonstrated that kinematic VIBF did not benefit skill acquisition, and did not improve task performance whether provided in isolation or coupled with visual feedback. VIBF had a deleterious effect on performance for some individuals. The study participants may have had difficulty integrating kinematic VIBF information into their control of a virtual myoelectric prosthetic arm.
